# (4-Hydr­oxy-2,5-dimethyl­phen­yl)phenyl­methanone

**DOI:** 10.1107/S1600536809039488

**Published:** 2009-10-03

**Authors:** Rodolfo Moreno-Fuquen, Leidy J. Valencia, Alan R. Kennedy, Denise Gilmour, R. H. De Almeida Santos

**Affiliations:** a Departamento de Química – Facultad de Ciencias, Universidad del Valle, Apartado 25360, Santiago de Cali, Colombia; b WestCHEM, Department of Pure and Applied Chemistry, University of Strathclyde, 295 Cathedral Street, Glasgow G1 1XL, Scotland; c Instituto de Química de São Carlos, Universidade de São Paulo, USP, São Carlos, SP, Brazil

## Abstract

The title compound, C_15_H_14_O_2_, was obtained by Friedel–Crafts acyl­ation between 2,5-dimethyl­phenol and benzoyl chloride in the presence of aluminium chloride as a catalyst. The dihedral angle between the benzene rings is 61.95 (4)°. In the crystal, O—H⋯O hydrogen bonding and C—H⋯O weak inter­actions lead to polymeric *C*(6), *C*(8) and *C*(11) chains along the *a*, *b* and *c*-axis directions, respectively.

## Related literature

For background information on the anti-fungal and anti-inflamatory biological activity of benzophenones, see: Naldoni *et al.* (2009 ▶); Selvi *et al.* (2003 ▶); Naveen *et al.* (2006 ▶). For 104 benzophenone mol­ecules, see: Cox *et al.* (2008 ▶). For hydrogen-bond motifs, see: Etter (1990 ▶).
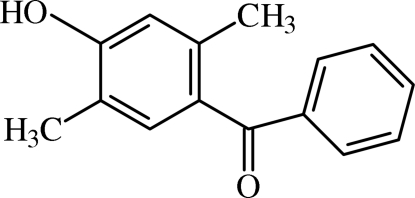


## Experimental

### 

#### Crystal data

C_15_H_14_O_2_
*M* *_r_* = 226.26
Orthorhombic, 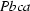

*a* = 12.1392 (10) Å
*b* = 8.1386 (7) Å
*c* = 23.665 (2) Å
*V* = 2338.0 (3) Å^3^
*Z* = 8
Mo *K*α radiation
μ = 0.08 mm^−1^
*T* = 123 K
0.25 × 0.12 × 0.05 mm

#### Data collection

Oxford Diffraction Gemini S diffractometer
Absorption correction: multi-scan (*CrysAlis CCD*; Oxford Diffraction, 2009 ▶) *T*_min_ = 0.904, *T*_max_ = 1.000
9067 measured reflections
2059 independent reflections
1061 reflections with *I* > 2σ(*I*)
*R*_int_ = 0.061

#### Refinement

*R*[*F* ^2^ > 2σ(*F* ^2^)] = 0.032
*wR*(*F* ^2^) = 0.058
*S* = 0.73
2059 reflections
158 parameters
H-atom parameters constrained
Δρ_max_ = 0.15 e Å^−3^
Δρ_min_ = −0.14 e Å^−3^

### 

Data collection: *CrysAlis CCD* (Oxford Diffraction, 2009 ▶); cell refinement: *CrysAlis CCD*; data reduction: *CrysAlis RED* (Oxford Diffraction, 2009 ▶); program(s) used to solve structure: *SHELXS97* (Sheldrick, 2008 ▶); program(s) used to refine structure: *SHELXL97* (Sheldrick, 2008 ▶); molecular graphics: *ORTEP-3 for Windows* (Farrugia, 1997 ▶) and *Mercury* (Macrae *et al.*, 2006 ▶); software used to prepare material for publication: *PARST95* (Nardelli, 1995 ▶).

## Supplementary Material

Crystal structure: contains datablocks I, global. DOI: 10.1107/S1600536809039488/hg2568sup1.cif

Structure factors: contains datablocks I. DOI: 10.1107/S1600536809039488/hg2568Isup2.hkl

Additional supplementary materials:  crystallographic information; 3D view; checkCIF report

## Figures and Tables

**Table 1:** Hydrogen-bond geometry (Å, °)

*D*—H⋯*A*	*D*—H	H⋯*A*	*D*⋯*A*	*D*—H⋯*A*
O2—H2⋯O1^i^	0.84	1.92	2.6973 (15)	154
C15—H15*B*⋯O1^ii^	0.98	2.62	3.352 (2)	132
C4—H4⋯O2^iii^	0.95	2.67	3.454 (2)	140

Symmetry codes: (i) 
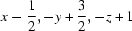
; (ii) 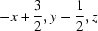; (iii) 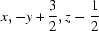.

**Fig. 1.**
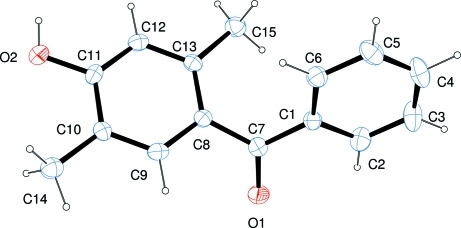
An ORTEP-3 (Farrugia, 1997) plot of the title (I) compound, with the atomic labelling scheme. The shapes of the ellipsoids correspond to 50% probability contours of atomic displacement and, for the sake of clarity, H atoms are shown as spheres of arbitrary radius.

**Fig. 2.**
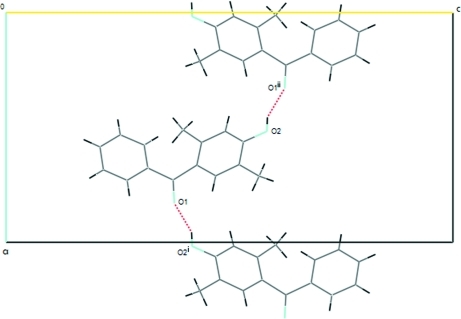
Part of the crystal structure of (I), showing the formation of C(8) chains running along [100] direction. Symmetry code: (i) x + 1/2, -y + 1/2, -z + 1; (ii) x - 1/2, -y + 1/2, -z + 1

**Fig. 3.**
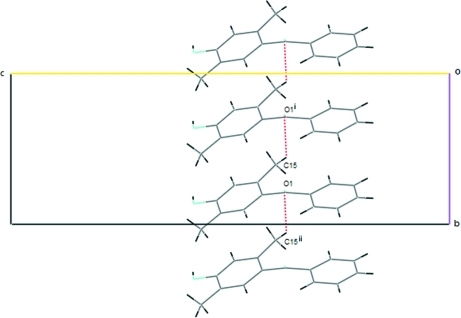
Part of the crystal structure of (I), showing the formation of C(6) chain running along [010]. Symmetry code: (i) -x + 3/2, y - 1/2, z; (ii) -x + 3/2, y + 1/2, z.

**Fig. 4.**
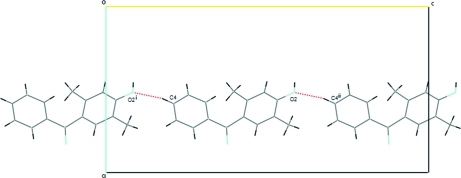
Part of the crystal structure of (I), showing the formation of C(11) chain running along [001]. Symmetry code: (i) x, -y + 3/2, z - 1/2; (ii) x, -y + 3/2, z + 1/2

## supplementary crystallographic information

### Comment

The title compound, C_15_H_14_O_2_, (4-Hydroxy-2,5-dimethyl-phenyl)-phenyl-methanone), (I), is part of a series of studies on benzophenone, which have been made in our research group. Benzophenone analogue systems show various anti-fungal and anti-inflamatory biological activities (Naldoni *et al.*, 2009 and Selvi *et al.*, 2003). The presence of various substituents in the benzophenone nucleus is essential to determining the quantitative structure-activity relationships of these systems. Some studies were carried out to show that methyl-substituted benzophenones exhibit anti-fungal properties (Naveen *et al.*, 2006). In order to present the molecular conformation of (I), to analyse the type of hydrogen-bonds formed in (I) and to study its supramolecular behavior, the title compound was synthesized. The molecular structure of the title compound is shown in Fig. 1. The bond lengths and bond angles of (I) are in good agreement with the standard values and correspond to those observed in (4-Hydroxy-3-methylphenyl)(4- methylphenyl)methanone (Naveen *et al.*, 2006). The two aromatic rings in the title structure form a dihedral angle of 61.95 (4)°. This value is greater than the value presented in the stable, orthorhombic form of unsubstituted benzophenone (54°) and follows the standard behavior of the majority of benzophenone molecules [104 benzophenone molecules, Cox *et al.*, 2008]. The title molecule is characterized by the formation of O—H···O hydrogen-bonds and other C—H···O weak interactions (Table 1, Nardelli, 1995). The strongest hydrogen bond O—H···O interaction is responsible for crystal growth in [100] direction. Indeed, in a first substructure, atom O2 in the molecule at (*x*, *y*, *z*) acts as hydrogen bond donor to carbonyl O1 atom in the molecule at (*x* - 1/2, -*y* + 1/2, -*z* + 1). The propagation of this interaction forms a C(8) (Etter, 1990) chain running along [100] direction (Fig. 2). In a second substructure, atom C15 in the molecule at (*x*, *y*, *z*) links with weak interaction to carbonyl O1 atom in the molecule at (-*x* + 3/2, *y* - 1/2, *z*). The propagation of this interaction forms C(6) continuous chains *via* C15—H15B···O1 and running along [010] direction (Fig. 3). Finally in a third sub-structure, atom C4 in the molecule at (*x*, *y*, *z*) links with weak interaction to hydroxyl O2 atom in the molecule at (*x*, -*y* + 3/2, *z* - 1/2). The propagation of this interaction forms C(11) continuous chains and running along [001] direction. All of these interactions in [100], [010] and [001] directions define the bulk structure of the crystal.

### Experimental

2,5-dimethylphenol (0.50 g, 4.10 mmol) was added to a solution of anhydrous aluminium chloride (0.40 g, 3.00 mmol) in dry dichloromethane (25 ml). The resulting solution was cooled and then a benzoyl chloride (0.80 g, 5.70 mmol) was slowly added at 0–5° C. After complete addition, the mixture was allowed to stir at room temperature for 0.5 h, and then it was heated up to 50° C for 1 h. The reaction mixture was poured onto ice (100 g) and conc. HCl (10 ml). The crude product was isolated by extraction with dichloromethane. The combined organic layers were washed with 10% aqueous NaOH, water, and then the solution was dried over Na_2_SO_4_ and it was evaporated at room temperature.

### Refinement

All H-atoms were located from difference maps and were positioned geometrically and refined using a riding model with C–H= 0.93–0.97 Å and *U*_iso_(H)= 1.2*U*_eq_(C).

### Crystal data

**Table:**

C_15_H_14_O_2_	*D*_x_ = 1.286 Mg m^−^^3^
*M**_r_* = 226.26	Melting point: 443.0(10) K
Orthorhombic, *P**b**c**a*	Mo *K*α radiation, λ = 0.71073 Å
Hall symbol: -P 2ac 2ab	Cell parameters from 1709 reflections
*a* = 12.1392 (10) Å	θ = 2.5–30.7°
*b* = 8.1386 (7) Å	µ = 0.08 mm^−^^1^
*c* = 23.665 (2) Å	*T* = 123 K
*V* = 2338.0 (3) Å^3^	Shard, colourless
*Z* = 8	0.25 × 0.12 × 0.05 mm
*F*(000) = 960	

### Data collection

**Table:**

Oxford Diffraction Gemini S diffractometer	2059 independent reflections
Radiation source: fine-focus sealed tube	1061 reflections with *I* > 2σ(*I*)
graphite	*R*_int_ = 0.061
ω scans	θ_max_ = 25.0°, θ_min_ = 3.1°
Absorption correction: multi-scan (*CrysAlis CCD*; Oxford Diffraction, 2009)	*h* = −12→14
*T*_min_ = 0.904, *T*_max_ = 1.000	*k* = −9→9
9067 measured reflections	*l* = −28→26

### Refinement

**Table:**

Refinement on *F*^2^	Secondary atom site location: difference Fourier map
Least-squares matrix: full	Hydrogen site location: inferred from neighbouring sites
*R*[*F*^2^ > 2σ(*F*^2^)] = 0.032	H-atom parameters constrained
*wR*(*F*^2^) = 0.058	*w* = 1/[σ^2^(*F*_o_^2^) + (0.0224*P*)^2^] where *P* = (*F*_o_^2^ + 2*F*_c_^2^)/3
*S* = 0.73	(Δ/σ)_max_ < 0.001
2059 reflections	Δρ_max_ = 0.15 e Å^−^^3^
158 parameters	Δρ_min_ = −0.14 e Å^−^^3^
0 restraints	Extinction correction: *SHELXL97* (Sheldrick, 2008), Fc^*^=kFc[1+0.001xFc^2^λ^3^/sin(2θ)]^-1/4^
Primary atom site location: structure-invariant direct methods	Extinction coefficient: 0.0019 (2)

### Special details

**Table:**

Geometry. All e.s.d.'s (except the e.s.d. in the dihedral angle between two l.s. planes) are estimated using the full covariance matrix. The cell e.s.d.'s are taken into account individually in the estimation of e.s.d.'s in distances, angles and torsion angles; correlations between e.s.d.'s in cell parameters are only used when they are defined by crystal symmetry. An approximate (isotropic) treatment of cell e.s.d.'s is used for estimating e.s.d.'s involving l.s. planes.
Refinement. Refinement of *F*^2^ against ALL reflections. The weighted *R*-factor *wR* and goodness of fit *S* are based on *F*^2^, conventional *R*-factors *R* are based on *F*, with *F* set to zero for negative *F*^2^. The threshold expression of *F*^2^ > σ(*F*^2^) is used only for calculating *R*-factors(gt) *etc*. and is not relevant to the choice of reflections for refinement. *R*-factors based on *F*^2^ are statistically about twice as large as those based on *F*, and *R*- factors based on ALL data will be even larger.

### Fractional atomic coordinates and isotropic or equivalent isotropic displacement parameters (Å^2^)

**Table:**

	*x*	*y*	*z*	*U*_iso_*/*U*_eq_	
O1	0.83004 (8)	0.77322 (14)	0.37654 (5)	0.0301 (3)	
O2	0.51698 (9)	0.86337 (14)	0.58360 (5)	0.0261 (3)	
H2	0.4559	0.8151	0.5849	0.039*	
C1	0.67652 (14)	0.8006 (2)	0.31719 (7)	0.0211 (4)	
C2	0.73020 (14)	0.7315 (2)	0.27099 (7)	0.0291 (5)	
H2A	0.7986	0.6768	0.2761	0.035*	
C3	0.68450 (16)	0.7422 (2)	0.21789 (7)	0.0370 (5)	
H3	0.7213	0.6946	0.1865	0.044*	
C4	0.58475 (16)	0.8226 (2)	0.21010 (8)	0.0363 (5)	
H4	0.5527	0.8278	0.1735	0.044*	
C5	0.53206 (15)	0.8948 (2)	0.25549 (7)	0.0306 (5)	
H5	0.4649	0.9524	0.2499	0.037*	
C6	0.57691 (14)	0.8834 (2)	0.30907 (7)	0.0248 (5)	
H6	0.5400	0.9317	0.3403	0.030*	
C7	0.72879 (13)	0.78937 (19)	0.37381 (7)	0.0210 (4)	
C8	0.66409 (13)	0.80471 (19)	0.42695 (7)	0.0186 (4)	
C9	0.71040 (13)	0.9034 (2)	0.46920 (7)	0.0209 (4)	
H9	0.7782	0.9572	0.4616	0.025*	
C10	0.66178 (13)	0.9261 (2)	0.52152 (7)	0.0185 (4)	
C11	0.56262 (13)	0.8428 (2)	0.53126 (7)	0.0195 (4)	
C12	0.51675 (13)	0.74203 (19)	0.49063 (7)	0.0210 (4)	
H12	0.4505	0.6849	0.4990	0.025*	
C13	0.56518 (13)	0.7218 (2)	0.43752 (7)	0.0189 (4)	
C14	0.71190 (13)	1.0335 (2)	0.56630 (7)	0.0270 (5)	
H14A	0.7342	0.9656	0.5986	0.040*	
H14B	0.6577	1.1150	0.5787	0.040*	
H14C	0.7766	1.0898	0.5509	0.040*	
C15	0.51105 (14)	0.6050 (2)	0.39635 (7)	0.0266 (5)	
H15A	0.4730	0.5180	0.4173	0.040*	
H15B	0.5673	0.5560	0.3719	0.040*	
H15C	0.4577	0.6653	0.3732	0.040*	

### Atomic displacement parameters (Å^2^)

**Table:**

	*U*^11^	*U*^22^	*U*^33^	*U*^12^	*U*^13^	*U*^23^
O1	0.0175 (6)	0.0449 (8)	0.0279 (7)	0.0032 (7)	−0.0010 (6)	0.0015 (7)
O2	0.0221 (7)	0.0330 (8)	0.0231 (7)	−0.0020 (6)	0.0038 (6)	0.0001 (6)
C1	0.0213 (10)	0.0219 (10)	0.0200 (10)	−0.0005 (9)	−0.0006 (9)	0.0020 (9)
C2	0.0321 (10)	0.0282 (12)	0.0269 (11)	0.0059 (9)	0.0025 (10)	0.0014 (9)
C3	0.0531 (13)	0.0374 (13)	0.0206 (11)	0.0084 (11)	0.0015 (11)	−0.0035 (9)
C4	0.0521 (14)	0.0336 (13)	0.0231 (12)	0.0027 (11)	−0.0110 (11)	0.0034 (10)
C5	0.0343 (12)	0.0256 (12)	0.0318 (12)	0.0013 (9)	−0.0102 (10)	0.0043 (10)
C6	0.0256 (10)	0.0246 (11)	0.0241 (11)	−0.0005 (9)	0.0002 (10)	−0.0013 (9)
C7	0.0231 (9)	0.0179 (10)	0.0219 (10)	0.0001 (8)	−0.0003 (9)	0.0007 (9)
C8	0.0176 (9)	0.0187 (10)	0.0196 (10)	0.0029 (8)	−0.0009 (9)	0.0019 (9)
C9	0.0172 (9)	0.0203 (10)	0.0253 (11)	−0.0006 (8)	−0.0015 (9)	0.0069 (9)
C10	0.0199 (10)	0.0173 (10)	0.0185 (10)	0.0035 (8)	−0.0030 (9)	0.0021 (8)
C11	0.0204 (10)	0.0207 (10)	0.0173 (10)	0.0063 (8)	0.0026 (9)	0.0037 (9)
C12	0.0170 (9)	0.0206 (11)	0.0253 (10)	−0.0004 (9)	0.0004 (8)	0.0049 (9)
C13	0.0173 (9)	0.0181 (10)	0.0215 (10)	0.0023 (8)	−0.0029 (8)	0.0006 (8)
C14	0.0252 (10)	0.0274 (11)	0.0283 (11)	−0.0007 (9)	0.0004 (9)	0.0027 (9)
C15	0.0267 (10)	0.0248 (11)	0.0283 (11)	−0.0032 (9)	−0.0006 (9)	−0.0002 (9)

### Geometric parameters (Å, °)

**Table:**

O1—C7	1.2377 (17)	C8—C13	1.400 (2)
O2—C11	1.3674 (18)	C8—C9	1.400 (2)
O2—H2	0.8400	C9—C10	1.384 (2)
C1—C2	1.391 (2)	C9—H9	0.9500
C1—C6	1.397 (2)	C10—C11	1.401 (2)
C1—C7	1.486 (2)	C10—C14	1.503 (2)
C2—C3	1.376 (2)	C11—C12	1.381 (2)
C2—H2A	0.9500	C12—C13	1.397 (2)
C3—C4	1.388 (2)	C12—H12	0.9500
C3—H3	0.9500	C13—C15	1.511 (2)
C4—C5	1.381 (2)	C14—H14A	0.9800
C4—H4	0.9500	C14—H14B	0.9800
C5—C6	1.383 (2)	C14—H14C	0.9800
C5—H5	0.9500	C15—H15A	0.9800
C6—H6	0.9500	C15—H15B	0.9800
C7—C8	1.488 (2)	C15—H15C	0.9800
			
C11—O2—H2	109.5	C10—C9—H9	118.5
C2—C1—C6	119.48 (15)	C8—C9—H9	118.5
C2—C1—C7	118.93 (15)	C9—C10—C11	116.70 (16)
C6—C1—C7	121.56 (15)	C9—C10—C14	122.40 (15)
C3—C2—C1	120.20 (16)	C11—C10—C14	120.90 (15)
C3—C2—H2A	119.9	O2—C11—C12	122.69 (15)
C1—C2—H2A	119.9	O2—C11—C10	115.98 (15)
C2—C3—C4	120.17 (17)	C12—C11—C10	121.29 (16)
C2—C3—H3	119.9	C11—C12—C13	121.78 (16)
C4—C3—H3	119.9	C11—C12—H12	119.1
C5—C4—C3	120.07 (17)	C13—C12—H12	119.1
C5—C4—H4	120.0	C12—C13—C8	117.69 (15)
C3—C4—H4	120.0	C12—C13—C15	118.11 (15)
C4—C5—C6	120.13 (17)	C8—C13—C15	124.11 (14)
C4—C5—H5	119.9	C10—C14—H14A	109.5
C6—C5—H5	119.9	C10—C14—H14B	109.5
C5—C6—C1	119.93 (16)	H14A—C14—H14B	109.5
C5—C6—H6	120.0	C10—C14—H14C	109.5
C1—C6—H6	120.0	H14A—C14—H14C	109.5
O1—C7—C1	118.54 (16)	H14B—C14—H14C	109.5
O1—C7—C8	119.27 (16)	C13—C15—H15A	109.5
C1—C7—C8	122.12 (14)	C13—C15—H15B	109.5
C13—C8—C9	119.56 (15)	H15A—C15—H15B	109.5
C13—C8—C7	124.28 (15)	C13—C15—H15C	109.5
C9—C8—C7	116.08 (15)	H15A—C15—H15C	109.5
C10—C9—C8	122.95 (16)	H15B—C15—H15C	109.5
			
C6—C1—C2—C3	−1.1 (3)	C13—C8—C9—C10	−1.2 (2)
C7—C1—C2—C3	−179.02 (16)	C7—C8—C9—C10	−178.15 (15)
C1—C2—C3—C4	0.2 (3)	C8—C9—C10—C11	0.8 (2)
C2—C3—C4—C5	1.3 (3)	C8—C9—C10—C14	−179.48 (16)
C3—C4—C5—C6	−1.8 (3)	C9—C10—C11—O2	178.50 (14)
C4—C5—C6—C1	0.9 (3)	C14—C10—C11—O2	−1.2 (2)
C2—C1—C6—C5	0.5 (3)	C9—C10—C11—C12	0.6 (2)
C7—C1—C6—C5	178.40 (16)	C14—C10—C11—C12	−179.08 (15)
C2—C1—C7—O1	24.8 (2)	O2—C11—C12—C13	−179.51 (14)
C6—C1—C7—O1	−153.09 (16)	C10—C11—C12—C13	−1.8 (2)
C2—C1—C7—C8	−158.14 (16)	C11—C12—C13—C8	1.4 (2)
C6—C1—C7—C8	24.0 (2)	C11—C12—C13—C15	178.17 (14)
O1—C7—C8—C13	−135.69 (17)	C9—C8—C13—C12	0.0 (2)
C1—C7—C8—C13	47.3 (2)	C7—C8—C13—C12	176.75 (15)
O1—C7—C8—C9	41.1 (2)	C9—C8—C13—C15	−176.54 (15)
C1—C7—C8—C9	−135.91 (16)	C7—C8—C13—C15	0.2 (2)

### Hydrogen-bond geometry (Å, °)

**Table:**

*D*—H···*A*	*D*—H	H···*A*	*D*···*A*	*D*—H···*A*
O2—H2···O1^i^	0.84	1.92	2.6973 (15)	154
C15—H15B···O1^ii^	0.98	2.62	3.352 (2)	132
C4—H4···O2^iii^	0.95	2.67	3.454 (2)	140

Symmetry codes: (i) *x*−1/2, −*y*+3/2, −*z*+1; (ii) −*x*+3/2, *y*−1/2, *z*; (iii) *x*, −*y*+3/2, *z*−1/2.
